# Water use of a multigenotype poplar short‐rotation coppice from tree to stand scale

**DOI:** 10.1111/gcbb.12345

**Published:** 2016-04-30

**Authors:** Jasper Bloemen, Régis Fichot, Joanna A. Horemans, Laura S. Broeckx, Melanie S. Verlinden, Terenzio Zenone, Reinhart Ceulemans

**Affiliations:** ^1^ Department of BiologyResearch Centre of Excellence on Plant and Vegetation EcologyUniversity of AntwerpUniversiteitsplein 1WilrijkB‐2610Belgium; ^2^ Université d'Orléans, INRA, LBLGCEA 1207F‐45067OrléansFrance

**Keywords:** bioenergy, evapotranspiration, poplar, sap flow, short‐rotation coppice, stand water balance

## Abstract

Short‐rotation coppice (SRC) has great potential for supplying biomass‐based heat and energy, but little is known about SRC's ecological footprint, particularly its impact on the water cycle. To this end, we quantified the water use of a commercial scale poplar (*Populus*) SRC plantation in East Flanders (Belgium) at tree and stand level, focusing primarily on the transpiration component. First, we used the AquaCrop model and eddy covariance flux data to analyse the different components of the stand‐level water balance for one entire growing season. Transpiration represented 59% of evapotranspiration (ET) at stand scale over the whole year. Measured ET and modelled ET were lower as compared to the ET of reference grassland, suggesting that the SRC only used a limited amount of water. Secondly, we compared leaf area scaled and sapwood area scaled sap flow (*F*_s_) measurements on individual plants vs. stand scale eddy covariance flux data during a 39‐day intensive field campaign in late summer 2011. Daily stem diameter variation (∆*D*) was monitored simultaneously with *F*_s_ to understand water use strategies for three poplar genotypes. Canopy transpiration based on sapwood area or leaf area scaling was 43.5 and 50.3 mm, respectively, and accounted for 74%, respectively, 86%, of total ecosystem ET measured during the intensive field campaign. Besides differences in growth, the significant intergenotypic differences in daily ∆*D* (due to stem shrinkage and swelling) suggested different water use strategies among the three genotypes which were confirmed by the sap flow measurements. Future studies on the prediction of SRC water use, or efforts to enhance the biomass yield of SRC genotypes, should consider intergenotypic differences in transpiration water losses at tree level as well as the SRC water balance at stand level.

## Introduction

Short‐rotation coppice (SRC) of fast‐growing and high‐yielding hardwood species as poplar and willow offers an important and environmentally sustainable way of producing heat and electricity from a renewable energy source (Herrick & Brown, 1967; Graham *et al*., 1992; Gustavsson *et al*., 1995; Berndes *et al*., 2003; Kauter *et al*., 2003; Aylott *et al*., 2008). Poplar SRC showed high biomass production rates of 10–15 t ha^−1^ yr^−1^ (Heilman *et al*., 1996; Trnka *et al*., 2008; Broeckx *et al*., 2012). However, there have been conflicting observations about the water use of SRC or its impact on the local water cycle. High‐yielding SRC has high water requirements (Hall & Allen, 1997; Hall *et al*., 1998; Allen *et al*., 1999; Meiresonne *et al*., 1999; Jassal *et al*., 2013; Navarro *et al*., 2014) potentially leading to negative effects on regional water resources (see references in Fischer *et al*., 2013). A number of – experimental and modelling – studies on evapotranspiration (ET, mm day^−1^) have argued that the water use of SRC is substantially higher than that of conventional agricultural crops or grasslands (see references in Dimitriou *et al*., 2009; Petzold *et al*., 2011). In contrast, other studies have reported that the water use rates of SRC are similar to those from agricultural crops and grasslands (Fischer *et al*., 2013), that is comparable to or lower than the reference crop evapotranspiration (ET_0_) (e.g. Meiresonne *et al*., 1999; Linderson *et al*., 2007; Migliavacca *et al*., 2009; Tricker *et al*., 2009).

It is expected that the transpiration component of ET (*E*_c_, mm day^−1^) is large as poplar species have high transpiration rates (Hall & Allen, 1997; Hall *et al*., 1998; Meiresonne *et al*., 1999; Kim *et al*., 2008). Simulated transpiration was 71% of ET for a poplar SRC in the Czech Republic (Fischer *et al*., 2013) and 66% of ET on a seasonal basis for a willow SRC in southern Sweden (Persson & Lindroth, 1994). Measurements of sap flow (*F*_s_, kg h^−1^) of individual trees scaled to the stand level are frequently used to quantify *E*_c_ for mature forest ecosystems (e.g. Oren *et al*., 1998; Schafer *et al*., 2002; Unsworth *et al*., 2004; Bovard *et al*., 2005; Tang *et al*., 2006; Oishi *et al*., 2008). Little is known, however, about the contribution of *E*_c_ to ET for SRC as only a limited number of studies combined plant‐level measurements with stand‐level water balance measurements or estimates of the water use of SRC. In addition, measurements of daily fluctuations in stem diameter (∆*D*) can provide complementary information on genotype‐specific tree water use as short‐term shrinkage and swelling are related to internal water storage dynamics (Zweifel *et al*., 2000, 2001; Larcher, 2003) and therefore changes in transpiration. As the first reports that stem dimensions change with changes in plant hydration (Fritts, 1961; Kozlowski & Winget, 1964; Impens & Schalck, 1965), short‐term high temporal resolution dendrometer measurements have been made on different forest species (see references in Zweifel *et al*., 2000; De Swaef *et al*., 2015), including on poplar genotypes grown under controlled conditions (Giovannelli *et al*. 2007). Field measurements of daily ∆*D* fluctuations, in combination with F_s_, might help to understand the dynamics in the contribution of *E*_c_ to ET for SRC and to identify unexplored intergenotypic differences in plant water use.

In this study, we monitored the water use of a poplar SRC in Flanders, Belgium, both at stand and at tree level. Our specific research hypotheses were as follows: (i) poplar SRC uses more water than a reference grassland under our specific conditions, (ii) *E*_c_ is the largest component of the stand water balance, and (iii) there are important intergenotypic differences in plant water use. For the entire growing season of 2011, we analysed the stand‐level water balance of the poplar SRC using the AquaCrop model (Hsiao *et al*., 2009; Raes *et al*., 2009; Steduto *et al*., 2009) complemented with eddy covariance measurements of ET. We further focused on tree‐level measurements of plant water use during an intensive field campaign performed during the same growing season. Using detailed tree‐level measurements of *F*_s_ and ∆*D*, we quantified the contribution of *E*_c_ to ET across three genotypes and we identified intergenotypic differences in plant water use.

## Materials and methods

### Study site and plant material

**Figure 1:**
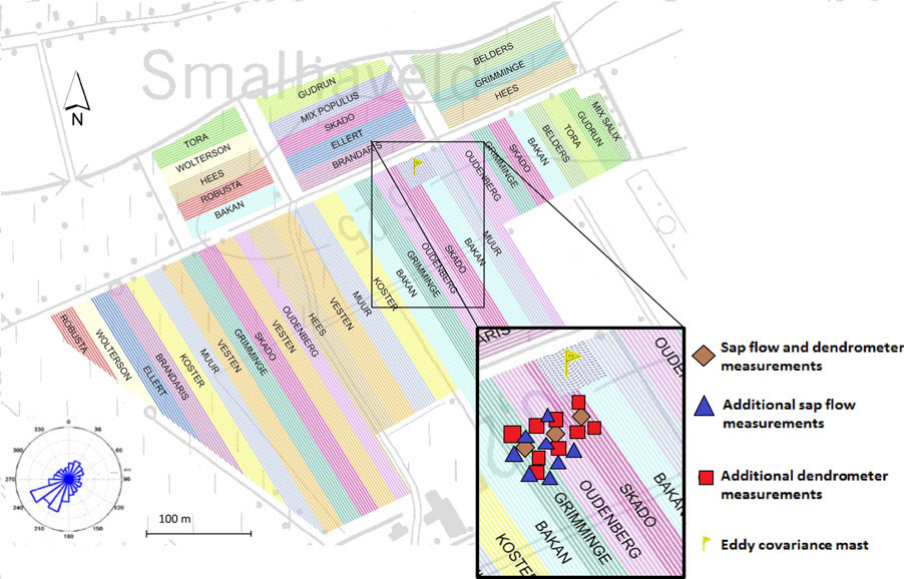
Map of the study site, indicating the location of the eddy covariance mast and the trees that were equipped with sap flow and/or dendrometer sensors during the 2011 field campaign. The wind rose for the period June 2010–December 2011 is also shown. The differently coloured wide bands indicate the mono‐genotypic blocks in the plantation.

Measurements were made in a commercial scale multigenotype SRC plantation, established in Lochristi, province East Flanders, Belgium (51°06′44″N, 3°51′02″E), at an elevation of 6.25 m above sea level. Long‐term average annual temperature at the site is 9.5 °C and the average annual precipitation is 726 mm, evenly distributed over the year. The soil has a loamy sand texture (clay content of 11% between 30 and 60 cm depth) with deeper clay‐enriched sand layers (~75 cm) and is classified as Anthrosol according to the World Reference Base for Soil Resources (Dondeyne *et al.,* 2015). On 7–10 April 2010, large replicated mono‐genotypic blocks were established over a total of 14.5 ha. Cuttings of 12 selected and commercially available poplar (*Populus*) genotypes (see Table 2 in Broeckx *et al*., 2012) were planted at a density of 8000 plants ha^−1^ (Fig. 1). Hardwood cuttings were planted in a double‐row design with alternating distances of 0.75 and 1.50 m between the rows and 1.1 m between the individuals within each row. The site was neither fertilized, nor irrigated. More information on the site, on the management and on soil characteristics is provided by Broeckx *et al*. (2012) and Verlinden *et al*. (2015).

An extendable eddy covariance and meteorological mast was positioned in the north‐eastern part of the plantation (Fig. 1) at the beginning of June 2010. Continuous ecosystem flux and microclimate measurements were then initiated (Zona *et al*., 2013a). The prevailing wind direction was from the south‐west (Fig. 1). Tree‐level sap flow (*F*_s_) and stem diameter variation (∆*D*) measurements were therefore performed within the flux footprint on the upwind side of the mast. These measurements were confined to a subset of the three genotypes closest to the mast (<15 m) characterized by a different parentage, namely Skado (parentage *Populus trichocarpa* T. & G.* *× *P. maximowiczii* A. Henry), Oudenberg (parentage *P. deltoides* Bartr. ex Marsh. × *P. nigra* L.) and Grimminge (parentage *P. deltoides* Bartr. ex Marsh. ×* P. trichocarpa* T. & G. ×* P. deltoides* Bartr. ex Marsh.) (Fig. 1). More details on the origin, the selection and the gender of these species are given by Broeckx *et al*. (2012). Stand‐level measurements were performed for the entire growing season of 2011, that is during the second growth year of the plantation and before the first coppice of the plantation (performed on 2–3 February 2012). Tree‐level measurements were made during an intensive field campaign from 19 August 2011 (day of the year, DOY 231) until 27 September 2011 (DOY 270). All tree‐level measurements were made on single stem trees as trees had not yet been coppiced.

### Stand‐level measurements and modelling

#### Climate variables

Climate variables were continuously recorded at the site: air temperature (*T*_air_) and relative humidity were recorded on the extendable mast at 5.4 m above the ground surface using Vaisala probes (model HMP 45C; Vaisala, Helsinki, Finland); these data were used to calculate vapour pressure deficit (VPD). Incoming photosynthetically active radiation (PAR, 400–700 nm) was recorded at the same height using a quantum sensor (model LI‐190; Li‐COR, Lincoln, NE, USA). Precipitation was recorded using a tipping bucket rain gauge (model 3665 R; Spectrum Technologies Inc., Plainfield, IL, USA). Soil water content (SWC) was measured diagonally in the 0–10 cm soil layers and horizontally at a specific depth of 40 cm next to the extendable eddy covariance mast using moisture probes (model TDR CS616; Campbell Scientific, Logan, UT, USA). Water table depth was recorded with a pressure transducer (model PDCR 1830; Campbell Scientific) installed in a pipe inserted into the ground to a depth of 1.85 m. Two data loggers (model CR5000 and CR1000; Campbell Scientific) recorded 30‐min averages for each environmental variable. If an instrument occasionally failed, the missing environmental variable (*T*_air_, relative humidity, or precipitation) was gap‐filled using data from nearby standard meteorological stations at 10 and 14 km from the research site. More information on the logging and the gap‐filling procedures for the environmental measurements has been published previously (Zona *et al*., 2013a,b).

#### Ecosystem evapotranspiration

Ecosystem level fluxes (of carbon, water and energy) were continuously monitored from the eddy covariance mast; in this study, only the water vapour fluxes are considered. High‐frequency (10 Hz) measurements of the three‐dimensional wind speed components were made using a sonic anemometer (model CSAT3; Campbell Scientific Inc.). Vertical wind velocity was combined with measurements from a closed‐path, fast‐response gas analyzer (model LI‐7000; Li‐COR) to measure the latent heat and to calculate evapotranspiration. Additionally, sensible heat fluxes were derived from vertical wind speed and sonic temperature measurements. The sonic anemometer and the inlets of the gas sample lines were positioned at 5.8 m above the ground for the period before 31 August 2011 (DOY 243) and afterwards raised to 6.6 m. Fluxes of latent heat (LE) were calculated using the EdiRe software (R. Clement, University of Edinburgh, UK; www.geos.ed.ac.uk/abs/research/micromet/EdiRe/) from high‐frequency data series divided into half‐hourly averaging periods. The two‐component rotation was applied to set mean lateral and vertical wind velocity components to zero, while the time delay between scalar and vertical wind velocity fluctuations was determined by cross‐correlation optimization. A filter rejected data using the following criteria: (i) more than one standard deviation for H_2_O vapour and (ii) for quality flags 9 as suggested by Foken & Wichura (1996) and Foken *et al*. (2004). The fluxes of LE were gap‐filled using the marginal distribution sampling (MDS) method (implemented in www.bgc-jena.mpg.de/~MDIwork/eddyproc/). This method is adopted by the FLUXNET community as a standardized gap‐filling technique. A detailed description of the ecosystem flux measurements at our site has been given by Zona *et al*. (2013a).

Additionally, we calculated reference crop evapotranspiration (ET_0_) for the site based on the Penman–Monteith method, as described by Allen *et al*. (1998). ET_0_ was integrated either daily or annually and compared with measured and modelled ET to assess the SRC water use relative to a reference crop.

#### Modelling of the stand water balance

We used the AquaCrop model to determine soil evaporation (*E*_soil_) as well as the *E*_c_ and ET components of the stand water balance over the growing season. AquaCrop is a crop water productivity and yield response model developed by the FAO that simulates daily biomass production and final crop yield (Table 1, Vanuytrecht *et al*., 2014). Briefly, *E*_c_ is calculated by multiplying ET_0_, determined using the Penman–Monteith method, with a crop coefficient (*K*c_Tr_) and a water stress coefficient (*K*_s_). *K*c_Tr_ is proportional to the fraction of the soil covered by the canopy (canopy cover, CC) and to *K*c_Tr,x_, the maximum crop transpiration coefficient for the specific crop relative to the grass reference surface. The *E*_soil_ also depends on ET_0_ and is further proportional to the fraction of the soil not covered by the canopy (1‐CC), to *K*_ex_ which is the maximum soil evaporation coefficient for a fully wet and unshaded soil surface, and to *K*_r_ which is the evaporation reduction coefficient that reduces transpiration when soil water content is low. In this study, we used the default value of 1 for both *K*_s_ and *K*_r_ as no water stress conditions were observed for the SRC plantation. All parameters relevant to the AquaCrop model, including the ones not mentioned above, are listed and explained in Table 1. Interception evaporation is not considered in the AquaCrop model. The ET is calculated as the sum of *E*_soil_ and *E*_c_ (Vanuytrecht *et al*., 2014). We performed a sensitivity analysis of the AquaCrop model for our site conditions. The results of this analysis were used to evaluate the effect of relative changes of a number of distributed parameters on the model outputs. Details on the simulated processes have been extensively documented in a set of publications at the model's release (Hsiao *et al*., 2009; Raes *et al*., 2009; Steduto *et al*., 2009) as well as in the FAO irrigation and drainage paper # 66 (Steduto *et al*., 2012) and in the reference manual (Raes *et al*., 2012).

**Table 1:** Definition of parameters, coefficients and variables of climate of stand‐ and tree‐level measurements, of sap flow scaling and of the AquaCrop model

Parameter/variable/coefficient	Symbol	Units	Value	Source
Climate variables				
Air temperature	*T*_air_	°C	Variable	
Vapour pressure deficit	VPD	kPa	Variable	
Photosynthetically active radiation	PAR	μmol m^−2^ s^−1^	Variable	
Soil water content	SWC	m^3^ m^−3^	Variable	
Stand‐level measurements				
Reference crop evapotranspiration	ET_0_	mm day^−1^	Variable	Allen *et al*. (1998)
Evapotranspiration	ET	mm day^−1^	Variable	
Transpiration component of evapotranspiration	*E*_c_	mm day^−1^	Variable	
Tree‐level measurements				
Stem diameter fluctuations	∆*D*	μm	Variable	
Maximum daily shrinkage	MDS	μm	Variable	
Day‐ and night‐time stem diameter fluctuation over time	∆*D*/∆*t*	μm h^−1^	Variable	
Sap flow scaling variables				
Average sapwood area for all trees equipped with dendrometers	A_s‐avg_	m^2^	Variable	
Sapwood area of the sample tree	*A*_s_	m^2^	Variable	
Ground surface area per tree	SA	m^2^	Table 2	Broeckx *et al*. (2015)
Sapwood area scaled transpiration component of evapotranspiration	*E*_c‐sapwood_	mm day^−1^	Variable	
Leaf area scaled transpiration component of evapotranspiration	*E*_c‐leaf_	mm day^−1^	Variable	
Genotype‐specific leaf area index	LAI	m^2^ m^−2^	Table 2	Broeckx *et al*. (2015)
Leaf area of the tree equipped with sap flow sensor	LA	m^2^	Table 2	
AquaCrop model				
Soil evaporation	*E*_soil_	mm yr^−1^	177	Raes *et al*. (2012)
Transpiration component of evapotranspiration	*E*_c_	mm yr^−1^	259	Raes *et al*. (2012)
Evapotranspiration	ET	mm yr^−1^	437	Raes *et al. (* 2012)
Reference crop evapotranspiration	ET_0_	mm yr^−1^	531	Allen *et al*. (1998)
Maximum soil evaporation coefficient for fully wet and not shaded soil surface	*K*_ex_	–	1.1	Raes *et al*.(2012)
Evaporation reduction coefficient	*K*_r_	–	1	Raes *et al*.(2012)
Green canopy cover	CC	%	Variable	Raes *et al*.(2012)
Actual canopy cover adjusted for micro‐advective effects	CC_star_	%	Variable	Raes *et al*.(2012)
Coefficient for maximum crop transpiration for well‐watered soil and complete canopy cover	*K*c_Tr,x_	–	Variable	Raes *et al*.(2012)
Crop transpiration coefficient	*K*_cTr_	–	1.2	Broeckx *et al*.(2015); Zenone *et al*. (2015)
Soil water stress coefficient	*K*_s_	–	1	Raes *et al*.(2012)
Soil surface covered by an individual seedling at 90% emergence	CC_s_	cm^2^	15	Field data (webcam images)
Number of plants per hectare	Den	plants ha^−1^	8000	Broeckx *et al*.(2015)
Initial canopy cover at time = 0	CC_0_	%	0.12	Raes *et al*.(2012)
Maximum canopy cover	CC_max_	m^2^ m^−2^	0.67	Broeckx *et al*.(2015)
Increase in canopy cover	CGC	Fraction day^−1^	0.058	Broeckx *et al*.(2015)
Decrease in canopy cover	CDC	Fraction day ^−1^	0.075	Broeckx *et al*.(2015)

### Tree‐level measurements

#### Sap flow measurements

The sap flow rate (*F*_s_, kg h^−1^) of individual trees was measured using the heat balance principle established in previous studies of SRC trees (e.g. Hinckley *et al*., 1994; Hall *et al*., 1998; Tricker *et al*., 2009; Petzold *et al*., 2011). *F*_s_ was monitored continuously during the entire field campaign, using three Dynamax sensors (model SG‐EX 25; Dynamax Inc., Houston, TX, USA), one on a tree of each genotype. The sensors were mounted at a height of 50 cm above the base of the stem. The sensors were thermally insulated from the environment with an insulation sleeve and several layers of aluminium foil wrapped around the sensor. *F*_s_ was calculated according to the standard procedure for heat balance sensors, described in detail by Sakuratani (1981) and Baker & Van Bavel (1987). In this study, we additionally tested for the heat storage effect (Steppe *et al*., 2005), but we did not observe early morning spikes in the *F*_s_ data. As the poplars were fast growing, we accounted for the increase in stem surface area during the *F*_s_ measurements using the increase in stem diameter recorded by high‐resolution dendrometers (see further below).

To validate the *F*_s_ measurements performed at 50 cm height and to account for variation in *F*_s_ among individuals, we also installed *F*_s_ sensors (models SG‐EX 16 and model SG‐EX 19; Dynamax Inc.) on four additional trees per genotype for the last twelve days of the intensive field campaign (15 September 2011–27 September 2011, DOY 258–270). Due to the limited number of sensors, these measurements were only performed on the Oudenberg and Grimminge genotypes and on a smaller stem section higher up the stem at approximately 2.5 m above the stem base. The basal stem diameter of the trees at the start of the additional F_s_ measurements ranged from 1.63 to 2.16 cm and from 1.51 to 1.84 cm for Oudenberg and Grimminge, respectively. Data from sap flow sensors were collected at 30‐s intervals with a data logger (model CR800; Campbell Scientific) and 30‐min averages recorded.

The relationship between *F*_s_ and VPD was analysed according to Tang *et al*. (2006) and Ewers *et al*. (2001) by fitting the following exponential saturation equation:

(1) $$F_{s}=a\left(1-e^{-b\mathrm{VPD}}\right)\times 24\frac{h}{d}$$

with *a* (kg day^−1^) and *b* (kPa^−1^) corresponding to the fitted coefficients, and 24 h day^−1^ corresponding to a time conversion factor. The relationship between *F*_s_ and PAR was analysed using a linear regression. For both analyses, *F*_s_ was summed per day and expressed relative to the daytime‐averaged VPD (with daytime defined as periods when PAR >5 μmol m^−2^ s^−1^) or the PAR summed per day.

#### Stem diameter measurements

Stem diameter fluctuation (∆*D*) was continuously measured using automatic point dendrometers (model ZN11‐O‐WP; Natkon, Hombrechtikon, Switzerland) installed with a ring‐shaped carbon frame at a height of 22 cm. Sensors were installed on four trees per genotype (12 sensors in total); one of these trees was also equipped with an *F*_s_ sensor as described above. Trees were selected to be representative of the whole range of stem diameters measured during an extensive inventory (*n* = 1742) performed in February 2011. Data from the dendrometers were collected at 30‐s intervals with a data logger (model CR800; Campbell Scientific) and 30‐min averages recorded. ∆*D* was expressed relative to the start of the measurement campaign, by setting the initial stem diameter to zero.

Changes in the stem water status were characterized by calculating the maximum daily shrinkage (MDS) as the difference between the maximum and minimum values of stem diameter during the day (Giovanelli *et al*. 2007). We also determined the day‐ and night‐time increases in stem diameter over time (∆*D*/∆*t*, with daytime defined as periods when PAR >5 μmol m^−2^ s^−1^) to determine different patterns in ∆*D* of trees of the different genotypes. We limited the combined analysis of *F*_s_, ET and ∆*D* to the first week of the measurement campaign (DOY 231–236) to clarify the links between the different variables. This period was characterized by a strong variation in VPD, as large dynamics in *T*_air_ were observed, leading to strong dynamics in *F*_s_, ET and ∆*D*.

#### Scaling of sap flow from tree to stand level

Two approaches were applied to scale *F*_s_ to canopy *E*_c_, which was then compared to ET. In a first approach, *F*_s_ was scaled to *E*_c_ by multiplying it by the ratio of the genotype‐specific leaf area index (LAI, m^2^ m^−2^) of the whole canopy to the total leaf area (LA, m^2^) of the individual tree equipped with a sap flow sensor:

(2) $$E_{c-\mathrm{leaf}}=F_{s}\frac{\mathrm{LAI}}{\mathrm{LA}}24\frac{h}{d}$$

with *E*_c‐leaf_ (mm day^−1^) corresponding to the leaf area scaled *E*_c_ and 24 h day^−1^ corresponding to a time conversion factor. LA was determined from genotype‐specific regressions relating leaf area with leaf length × leaf width (*R*² ≥0.99 for all genotypes) for a minimum sample of 25 leaves spanning the whole leaf size range from trees neighbouring those equipped with sap flow sensors. Harvested leaves were scanned and analysed using ImageJ software (NIH, Bethesda, MD, USA). Leaf length and width of all leaves on the trees equipped with sap flow sensors were measured before (i.e. 10 August 2011, DOY 222) and after the intensive field campaign (i.e. 28 September 2011, DOY 271) to account for the change in LA (Table 2). LAI was monitored for different locations in the study site for three occasions during the period July–September 2011 [on 22 July (DOY 203), on 2 September (DOY 245) and on 23 September (DOY 266)] using cross‐calibrated plant canopy analyzers (models LAI‐2000 and LAI‐2200; Li‐COR). We selected data from the measurements performed closest to the *F*_s_ sensors, which we assumed to best represent the LAI in the footprint of the eddy covariance measurements (Table 2). More information on the LAI measurements has been published previously (Broeckx *et al*., 2012).

**Table 2:** Parameters used for scaling sap flow rate to canopy transpiration during the field campaign from 19 August (DOY 231)–27 September (DOY 270) 2011, according to a leaf‐based approach [leaf area (LA) or genotype‐specific leaf area index (LAI)] and a stem‐based approach [ground surface area per tree (SA)] for three poplar genotypes

Genotype	LA (m²)		LAI (m^2^ m^−2^)			SA (m^2^)
	DOY 222	DOY 271	DOY 203	DOY 245	DOY 266	
Skado	1.2	1.4	1.9	2.1	2.1	1.2
Oudenberg	1.2	1.5	1.6	1.9	2.1	1.3
Grimminge	1.8	2.0	1.6	2.4	2.2	1.4

DOY, day of the year. See Materials and Methods for additional description.

In a second approach *F*_s_ was scaled to *E*_c_ by multiplying it by the ratio of the average sapwood area for all trees equipped with dendrometers (*A*_s‐avg_, m^2^) to the sapwood area of the sample tree (*A*_s_, m^2^) and per unit of average ground surface area per tree (SA, m^2^):

(3) $$E_{c-\mathrm{sapwood}}=F_{s}\frac{A_{s-\mathrm{avg}}}{A_{s}\text{SA}}24\frac{h}{d}$$

with *E*_c‐sapwood_ (mm day^−1^) corresponding to the sapwood area scaled *E*_c_ and 24 h day^−1^ corresponding to a time conversion factor. Both *A*_s_ and *A*_s‐avg_ were estimated using the dendrometer data, assuming that for these young trees the entire stem consisted of functional sapwood except for a small fraction of bark tissue. SA was estimated for each genotype based on the spacing and the consistent stocking of trees in the mono‐genotypic block design of the site (Table 2). Finally, both *E*_c‐leaf_ and *E*_c‐sapwood_ were averaged for the three genotypes and summed per day for comparison with the daily sums of ecosystem ET.

### Statistical analysis

For the stand‐level measurements, we used Pearson correlation and linear regression analysis to determine correlations and regressions between measured and modelled data. For the intensive field campaign, the rate of change in diameter, ∆*D*/∆*t*, was analysed using a repeated‐measures anova model with genotype (*n* = 3) and night‐ or daytime period (*n* = 11) as fixed factors and individual tree (*n* = 4) treated as random factor. A similar model was used to analyse MDS; however, data were confined to the daytime period (*n* = 5). The Akaike information criterion correcting for small sample sizes (AICc) was used to determine the covariance structure that best estimated the correlation among individual trees over time. Treatment means were compared using Fisher's least significance difference test. anova analyses were performed using the mixed model procedure (PROC MIXED) of sas (Statistical Analysis System, Cary NC, USA) with *α *= 0.05.

## Results

### Environmental conditions during the measurement campaign

**Figure 2:**
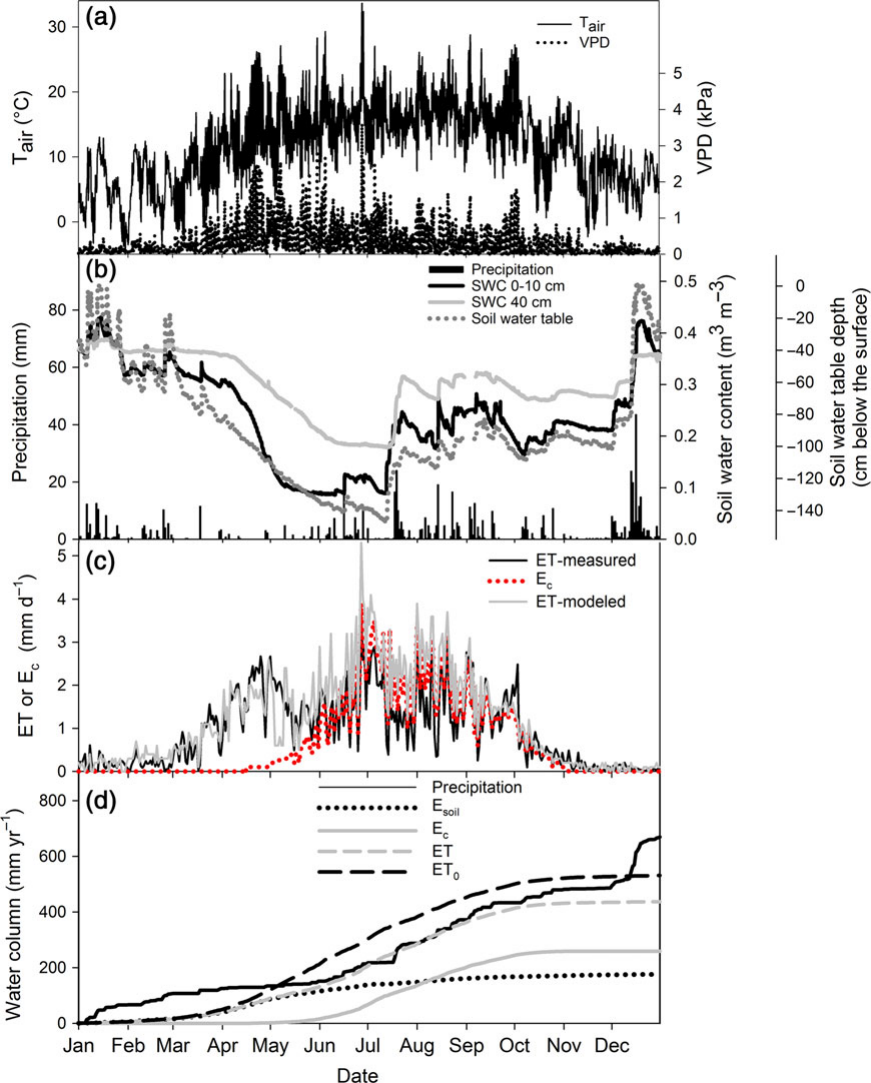
Time course of meteorological variables of measured and modelled evapotranspiration (ET) and of the cumulative evaporative components during the year 2011. (a) Air temperature (*T*_air_; solid line) and vapour pressure deficit (VPD; dotted line); (b) daily summed precipitation (black bars), soil water content (SWC) measured at 0–10 cm (black solid line) and 40 cm (grey solid line) depth, and water table depth (dark grey dotted line); (c) daily measured ET (solid black line), modelled ET (solid grey line) and modelled canopy transpiration (*E*_c_, dotted red line). (d) Cumulative precipitation (black solid line), soil evaporation (*E*_soil_, black dotted line), *E*_c_ (grey solid line), ET (grey dashed line) and reference evapotranspiration (ET_0_, black dashed line).

Variable weather conditions were experienced during the measurement campaign (Fig. 2a,b). For instance, VPD (Fig. 2a) varied strongly during the measurement period leading to a range of *E*_c_ and ET rates (Fig. 2c). The maximum VPD of 3.6 kPa was observed on 27 June 2011 (DOY 178), which coincided with maximum modelled ET and *E*_c_, and measured ET. Precipitation patterns were dynamic as relatively dry periods alternated with periods of rainfall (Fig. 2b). In response to precipitation events, SWC measured at 0–10 cm depth increased, together with a less pronounced increase in SWC at 40 cm depth and a rising water table (Fig. 2b).

### Yearly stand water balance

Both modelled and measured daily ET showed similar dynamics (Pearson's correlation coefficient: 0.861) that were strongly related to changes in VPD (Fig. 2c). Modelled daily *E*_c_ started to increase from mid‐April onwards up to a maximum of 3.9 mm day^−1^ at 27 June 2011. At the end of the growing season, *E*_c_ decreased from late September onwards, as leaf fall started around that period. The average modelled daily *E*_c_ for the growing season was 1.3 mm day^−1^.

Summed over 2011 modelled ET was 437 mm, which was 87 mm higher than measured ET (350 mm) but still 94 mm lower than ET_0_ (531 mm, Fig. 2d). Cumulative *E*_c_ was 259 mm, representing 59% of ET over the whole year, as derived from the modelled data. When considering the actual growing season (from mid‐April to late September), *E*_c_ represented 69% of ET. Total modelled *E*_soil_ was smaller than *E*_c_ (177 mm) and accounted for 41% of the total ET. Total cumulative measured precipitation was 669 mm, which was higher than the total ET and ET_0_. Run‐off at our site was negligible for the stand water balance. The remainder of the precipitation was lost to groundwater leaching. The results from the sensitivity analysis of the model parameters showed that CGC had the largest impact on modelled *E*_soil_, *E*_c_ and ET followed by *K*c_Tr,x_ (Table 3). In contrast, CDC had a limited impact on modelled *E*_soil_, *E*_c_ and ET. Overall, the deviation in model output observed during the sensitivity analysis ranged from −22.4% to +14.1%. Changes in parameter values had the largest impact on *E*_c_, except for the parameter *K*_ex_ which only impacted *E*_soil_.

**Table 3:** Sensitivity analysis of the parameters used to model soil evaporation (*E*_soil_), the transpiration component of evapotranspiration (*E*_c_) and evapotranspiration (ET) for the multigenotype SRC over the 2011 growing season. The analysis evaluates the effect of changes in a range of site‐specific realistic parameter values on the model output. Given are the % deviation at the minimum parameter value (min % deviation), the % of deviation at the maximum parameter value (max % deviation) and the total (total % deviation) deviation of modelled *E*_soil_, *E*_c_ and ET relative to the base value used in the study

	CC_max_	CC_0_	*K*_ex_	*K*c_Tr,x_	CGC	CDC
Value used in study	0.67	0.12	1.1	1.2	0.058	0.075
Minimum value	0.57	0.1	1	1	0.048	0.065
Maximum value	0.77	0.4	1.2	1.3	0.068	0.085
Min % deviation *E*_soil_	+5.0	+0.6	−6.3	+0.4	+8.8	−0.3
Max % deviation *E*_soil_	−4.9	−4.5	+6.1	−0.2	−5.7	+0.3
Min % deviation *E*_c_	−10.8	−1.5	0.0	−16.7	−22.4	+0.9
Max % deviation *E*_c_	+9.8	+11.3	0.0	+8.3	+14.1	−0.7
Min % deviation ET	−4.2	−0.6	−2.6	−9.6	−9.4	+0.4
Max % deviation ET	+3.7	+4.7	+2.5	+4.7	+5.9	−0.3
Total % deviation for *E*_soil_	9.9	5.1	12.4	0.7	14.4	0.6
Total % deviation for *E*_c_	20.6	12.8	0.0	25.0	36.5	1.6
Total % deviation for ET	7.9	5.4	5.1	14.3	15.3	0.7

CC_max_, maximum canopy cover; CC_0_, initial canopy cover at time = 0; *K*_ex_, maximum soil evaporation coefficient for fully wet and not shaded soil surface; *K*c_Tr,x_, crop transpiration coefficient; CGC, increase in canopy cover; CDC, decrease in canopy cover.

### Sap flow

**Figure 3:**
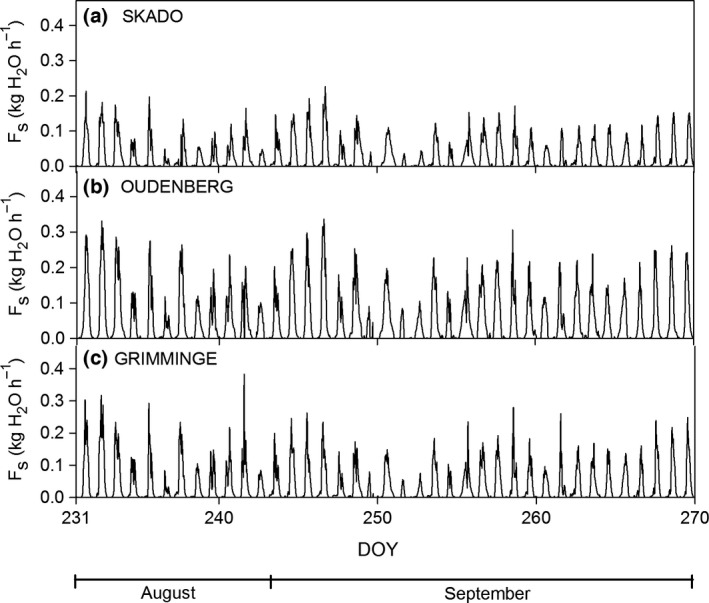
Time course of sap flow (*F*_s_) monitored using heat balance sensors during the intensive field campaign [19 August (DOY 231)–27 September (DOY 270) 2011] for genotypes (a) Skado, (b) Oudenberg and (c) Grimminge. DOY, day of the year.

The highest F_s_ rates were observed for Oudenberg (Fig. 3b), as compared to Skado (Fig. 3a) and Grimminge (Fig. 3c), with a maximum *F*_s_ rate of 0.3 kg h^−1^ on DOY 247. Skado had the lowest *F*_s_ rates with a maximum *F*_s_ rate of 0.2 kg h^−1^; this occurred on the same day as the maximum *F*_s_ for Oudenberg. The additional *F*_s_ measurements performed for both Oudenberg and Grimminge between 15 September (DOY 258) and 27 September (DOY 270) 2011 confirmed the higher *F*_s_ rates of Oudenberg as compared to Grimminge (data not shown). *F*_s_ measured with these sensors installed higher up the tree varied in synchrony with the observed patterns in *F*_s_ obtained with sensors installed at the stem base. For Oudenberg, *F*_s_ at the stem base was within the range of *F*_s_ rates measured during the additional campaign. For Grimminge, the daytime *F*_s_ measured higher up the stem was on average 0.04 kg h^−1^ lower as compared to the *F*_s_ measured at 50 cm.

**Figure 4:**
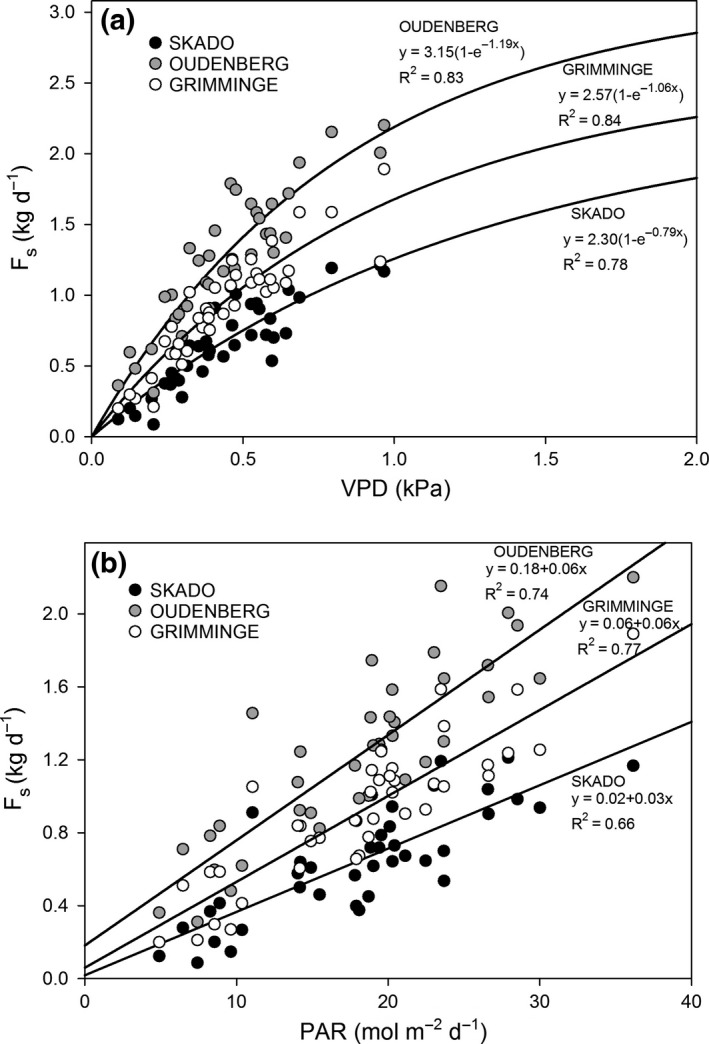
Daily summed tree sap flow (*F*_s_) measured at stem base for genotypes Skado (black dots), Oudenberg (grey dots) and Grimminge (white dots) both as a function of (a) daytime average VPD and (b) photosynthetically active radiation (PAR). Lines are exponential saturation curves [Eqn.: *y* = *a*(1 −*e* ^−*b* VPD^)] and linear curves (Eqn.: *y* = *ax* + *b*) in (a) and (b), respectively. VPD used to calculate daytime averages was selected when PAR >5 μmol m^−2^ s^−1^.

Daily sums of *F*_s_ were significantly correlated with daytime‐averaged VPD (Fig. 4a, *P* < 0.001). The maximum *F*_s_ rate, estimated by coefficient ‘*a*’ in Eqn (2), was higher for Oudenberg (3.2 kg h^−1^) than for Grimminge (2.6 kg h^−1^) and Skado (2.3 kg h^−1^) (Fig. 4a). A similar genotypic difference was observed for the *F*_s_ – PAR regression (Fig. 4b).

### Daily stem diameter variation

**Figure 5:**
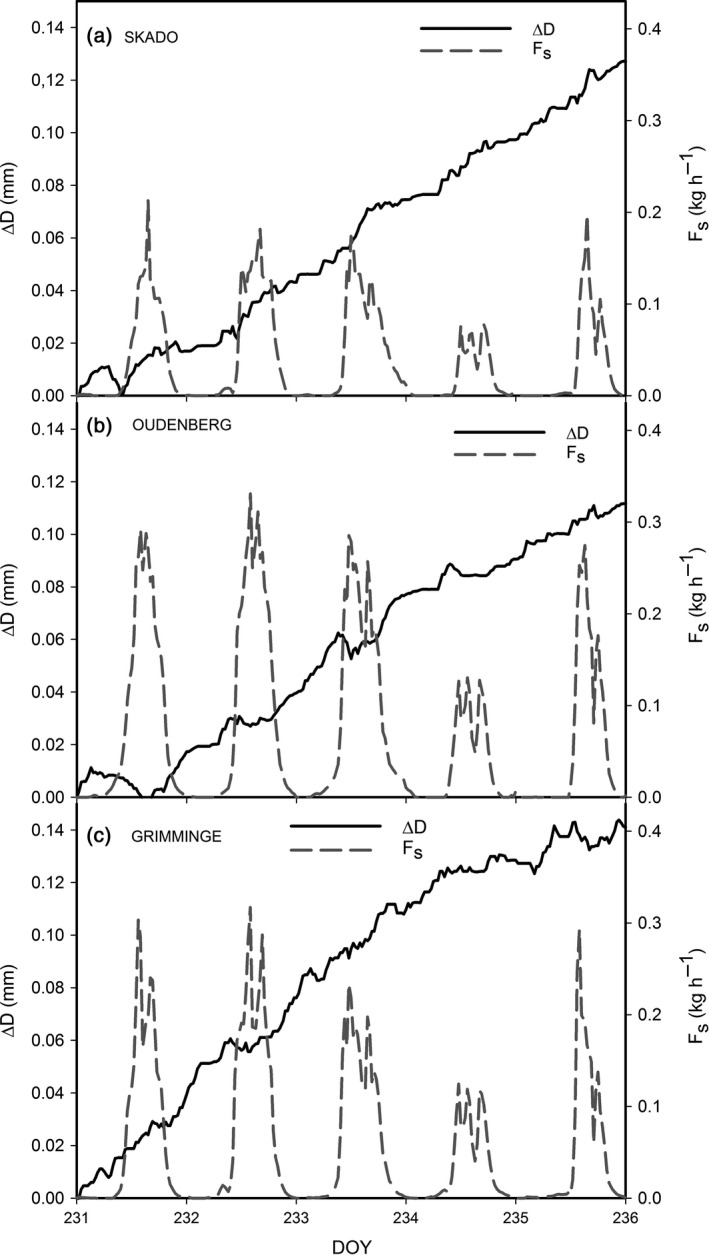
Time course of short‐term stem diameter variations (∆*D*; solid line) and of sap flow (*F*_s_; dashed line) both measured at stem base for the genotypes (a) Skado, (b) Oudenberg and (c) Grimminge during the period 19 August (DOY 231)–23 August (DOY 236) 2011. ∆*D* is expressed relative to the start of the measurement campaign, by setting the initial stem diameter to zero. DOY, day of the year.

Growth during the 39‐day measurement campaign significantly differed among the three genotypes. Average (±SE) stem diameter increase during the intensive field campaign was significantly higher (*P* < 0.01) for Skado (0.8 ± 0.1 mm) as compared to Oudenberg (0.4 ± 0.1 mm) and Grimminge (0.4 ± 0.1 mm). More interestingly, daily ∆*D* variations were observed for all three genotypes (Fig. 5) as trees tended to shrink during daytime when *F*_s_ was high and swelled during the night when they replenished their water reserves. The Skado tree, equipped with both *F*_s_ and dendrometer sensors, did not show a significant shrinkage during the day (except at the onset of *F*_s_ during DOY 231, Fig. 5a). The Oudenberg tree, and to a lesser extent the Grimminge tree, significantly shrank during days with high *F*_s_ rates (Fig. 5b,c).

**Figure 6:**
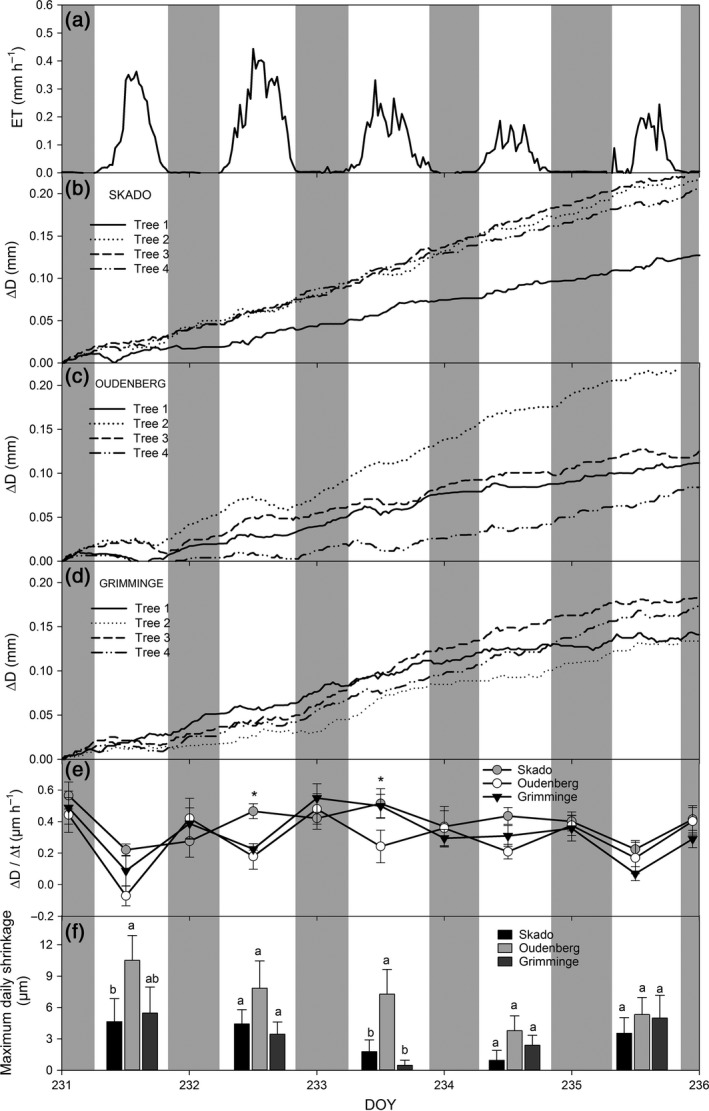
Time course of evapotranspiration (ET) measured using eddy covariance (a), of stem diameter variations (∆*D*) (b–d), of day‐ and night‐time stem diameter variation over time (∆*D*/∆*t*;* n* = 4 per genotype) (e) and of daytime maximum daily shrinkage (MDS) (f). (a) ET; (b–d) ∆*D* for four trees per genotype; and (e) ∆*D*/∆*t* for genotypes Skado (grey dot), Oudenberg (white dot) and Grimminge (black triangle) during the period 19 August (DOY 231)–23 August (DOY 236) 2011. ∆*D*/∆*t* was calculated as the difference in diameter at the start and at the end of a day‐ or night‐time period divided by the duration of that period. For data selection, daytime was taken as the period when photosynthetically active radiation (PAR) >5 μmol m^−2^ s^−1^. Asterisks indicate significant (*P* < 0.05) differences in ∆*D*/∆*t*. (f) MDS for genotypes Skado (black bar), Oudenberg (grey bar) and Grimminge (dark grey bar). MDS was calculated as the difference in maximum and minimum stem diameter during the daytime. Different letters indicate significant (*P* < 0.05) differences in MDS among genotypes. Grey shaded areas represent night‐time periods. DOY, day of the year.

Similar patterns in ∆*D* were observed for the other trees equipped with dendrometers (Fig. 6). Regardless of the genotype, stem diameter growth was observed for all trees of the different genotypes (Fig. 6b–d), but differences in the day‐ and night‐time ∆*D* were observed among genotypes (Fig. 6e). The change in ∆*D*/∆*t* for both day‐ and night‐time confirmed the intergenotypic differences in tree water use as observed with F_s_ measurements. Oudenberg showed a consistently higher ∆*D*/∆*t* during the night as compared to the daytime (Fig. 6e). Genotypes Grimminge and in particular Skado showed a larger variability in the day‐ and night‐time patterns of ∆*D*/∆*t*, resulting in significant differences in daytime ∆*D*/∆*t* among genotypes on DOY 232 (*P* = 0.0447) and DOY 233 (*P* = 0.0458).

Significant differences in MDS (*P* < 0.01) were observed among the three genotypes (Fig. 6f). Oudenberg showed the highest MDS (maximum 10.5 μm), significantly higher than Skado (maximum 4.7 μm) and Grimminge (maximum 5.5 μm) on both DOY 231 (*P* < 0.05) and DOY 233 (*P* < 0.05). Small differences in MDS among the genotypes were observed during the days when lower ET (Fig. 6a) and *F*_s_ (Fig. 5) occurred, that is DOY 234 and 235.

### Scaling of sap flow from tree to stand level

**Figure 7:**
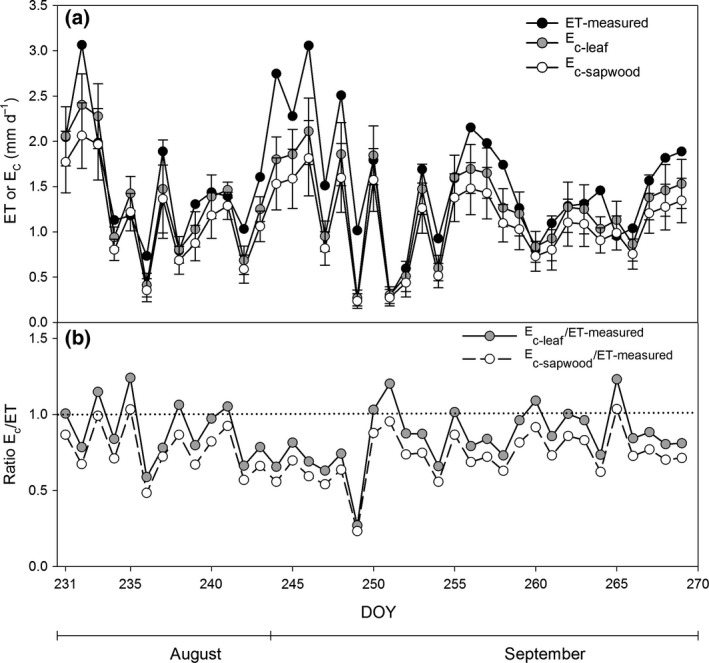
Time course of (a) daily summed evapotranspiration (ET) and daily summed canopy transpiration (*E*_c_) estimated using sapwood area‐based (*E*_c‐sapwood_) and leaf area‐based (*E*_c‐leaf_) scaling of sap flow, and (b) the ratio of *E*_c‐sapwood_ or *E*_c‐leaf_ over ET during the intensive field campaign (19 August (DOY 231)–27 September (DOY 270) 2011). (a) ET (black dots), *E*_c‐leaf_ (grey dots) and *E*_c‐sapwood_ (white dots). Error bars represent standard errors (SEs). (b) Ratio of *E*_c‐leaf_ over ET (grey dots) and *E*_c‐sapwood_ over ET (white dots). The dotted line indicates a ratio equal to one which implies that *E*_c‐sapwood_ or *E*_c‐leaf_ is equal to ET. DOY, day of the year.

Scaling of *F*_s_ to *E*_c‐sapwood_ and *E*_c‐leaf_ resulted in daily average canopy transpiration rates of 1.1 and 1.3 mm day^−1^, respectively (Fig. 7a). Total *E*_c‐sapwood_ and *E*_c‐leaf_ for the measurement campaign were 43.5 and 50.3 mm, respectively. Both values were lower than the total ET from eddy covariance, that is 59.9 mm. For most of the days during the field campaign, ET was higher than both *E*_c‐sapwood_ and *E*_c‐leaf_, resulting in a ratio of *E*_c_/ET lower than unity (Fig. 7b). Assuming that ET and *E*_c_ were comparable, transpiration accounted for 74% and 86% of total ET, using average *E*_c‐sapwood_/ET and *E*_c‐leaf_/ET averaged over the period of the intensive field campaign as an estimate of the contribution of *E*_c_ to ET, respectively. Overall, the leaf area‐based approach tended to overestimate *E*_c_ relative to ET (i.e. *E*_c_/ET >1) more than the sapwood area‐based scaling of *F*_s_ to *E*_c_. The differences between ET and *E*_c_ resulted from *E*_soil_ and from the transpiration of understory weed vegetation, accounting for 26% and 14% of total ET when estimating *E*_c_ using the sapwood and leaf area‐based approach, respectively.

## Discussion

### Stand water balance

It has been argued that SRCs might have a strong impact on the regional water cycle. The stand water balance analysis at our site suggests that the impact of the SRC on the regional water cycle was not negative. First, our site was not water limited, as precipitation was around 53% and 26% higher than annual ET and ET_0_, respectively. Secondly, for the year 2011, ET of our poplar SRC was around 18% lower as compared to ET_0_, suggesting that the site used less water as compared to a reference grassland. Thirdly, an increase in the ecosystem water use efficiency over the year 2011 (reported earlier by Broeckx *et al*., 2014a, b) suggested that the poplars at our site could reduce the transpiration water loss per unit of fixed carbon by regulating stomatal opening. Caution is advised in generalizing these results to other SRCs. While studies on SRC water use in the Czech Republic (Fischer *et al*., 2013), in Mongolia (Hou *et al*., 2010) and in the USA (Nagler *et al*., 2007) showed similar results, almost a same number of studies (see references in Fischer *et al*., 2013) have shown that SRCs across the globe consume more water as compared to traditional agricultural crops or grasslands. Therefore, site location, local climatic conditions, species considered and the age of the plantation are important factors that determine the actual SRC water use and stand water balance (IEA‐Bioenergy, 2011).

In line with results from previous studies (e.g. Persson & Lindroth, 1994; Fischer *et al*., 2013), both the modelling approach of *E*_c_ and the scaling approach of *F*_s_ measurements to stand level showed that *E*_c_ represented the largest component of ET. The average daily *E*_c_ values for our site (1.3, 1.1 and 1.3 mm day^−1^, for modelled, sapwood and leaf area scaled *E*_c_, respectively) were within the lower end of the range of 1–8 mm day^−1^ reported for poplar stands of different genotypes, stand age and geographic locations in temperate climate zones (Meiresonne *et al*., 1999). For an irrigated *P. trichocarpa* × *P. deltoides* plantation in the Pacific Northwest of the USA, an average *E*_c_ of 4 mm day^−1^ was observed (Kim *et al*., 2008). Sap flow – measured with the same heat balance principle as in the present study – provided a growing season average *E*_c_ of 2.2 mm day^−1^ for a *P*. *maximowiczii* × *P*. *nigra* plantation in southern Germany (Petzold *et al*., 2011). Both last mentioned studies on poplar *E*_c_ were performed for either single or multishoot stands, while the 2‐year‐old trees of our site were still single stemmed and had not reached full canopy closure yet (Broeckx *et al*., 2012). Moreover, the trees considered in previous studies were older and probably had a larger leaf and sapwood conducting area than the trees at our site; this partly explains the higher stand transpiration values found in the literature.

Our *E*_c_/ET estimates based on modelling (69%) and *F*_s_ scaling (74% and 86%) were consistent with other published findings for different SRC cultures. A recent review on bioenergy water requirements showed that the average absolute water use of tree‐based SRC was 618 mm per year, with 75% of this amount directly transpired by the trees (King *et al*., 2013). Likewise, a literature survey (Fischer *et al*., 2013) and simulations for a ‘hypothetical’ SRC (Grip *et al*., 1989) showed that transpiration contributed on average to 80% and 71% of the seasonal ET of SRC, respectively. For an experimental pine and switchgrass intercrop forestry system in North Carolina (USA), transpiration modelled over 3 years was on average 62% of ET (Albaugh *et al*., 2014).

### Uncertainties related to estimating stand‐level water use

A number of uncertainties may arise when scaling up data from the individual tree to the stand and levels or when modelling stand water balance components. These uncertainties were, however, considered to be minimized in our case for the following reasons.

Uncertainties can be associated with the eddy covariance measurements of ecosystem fluxes (e.g. Baldocchi, 2003). However, for our site, the energy balance closure (based on the assessment of net radiation, latent and sensible heat flux densities, soil heat flux density and energy storage) was 93% in 2011 (Zona *et al*., 2013a). This value therefore shows the good performance of the eddy covariance system in measuring fluxes at our site during the year 2011 when our measurements were performed. In addition, potential mismatches between spatial footprints of *F*_s_ and ET measurements, which depend on the wind direction, were minimized by measuring *F*_s_ close to the mast and on the upwind side for the prevailing wind direction.

Uncertainties are also associated with the methods used for scaling *F*_s_. On the one hand, for the sapwood area‐based scaling of *F*_s_, we assumed that the whole stem cross section consisted of conducting sap wood; however, this approach was successfully used for scaling *F*_s_ to stand level for a *P. nigra* × *P. maximimowczii* SRC in Wisconsin, USA (Zalesny *et al.,* 2006) such that uncertainties were probably limited for this approach. On the other hand, uncertainties were clearly associated with the LA scaling of *E*_c_, leading to a more frequent overestimation of *E*_c_ than the sapwood area scaling approach (i.e. *E*_c_/ET frequently higher than 1). These uncertainties likely resulted from both the genotype‐specific allometric reconstruction of LA and the heterogeneity in LAI around the mast. Therefore, at our site, a sapwood area scaling approach was preferable for scaling *F*_s_ to *E*_c_. Future studies should combine different techniques across different spatial and temporal scales and a range of environmental conditions (Fischer *et al*., 2013).

In addition to the uncertainties depending on the approach used for scaling *F*_s_, *F*_s_ can also vary considerably among individuals of a given genotype, which means that replicates are needed to give an accurate estimate of the mean flux (Oren *et al*., 1998; Oishi *et al*., 2008). As *F*_s_ measurements in our study were limited to only 39 days, we used the AquaCrop model to estimate *E*_c_ for the whole growing season. However, the choice of the model parameter values to determine the yearly stand water balance was also prone to uncertainty. For instance, model parameters used to describe canopy development will have an impact on both the transpiration and the soil evaporation component of the stand water balance. To this end, an additional model sensitivity analysis could be used to evaluate the effect of parameter changes on the model outputs, as performed for the AquaCrop model in our study.

### Intergenotypic differences in tree water use

Previously reported measurements at this site, made in the same year 2011 (Broeckx *et al*., 2014a), revealed differences in stomatal conductance among genotypes to be strongly related to differences in F_s_ observed during our study. According to Broeckx *et al*. (2014a), Oudenberg showed the highest average stomatal conductance (459 mmol s^−1^ m^−2^), while Grimminge (319 mmol s^−1^ m^−2^) and in particular Skado (255 mmol s^−1^ m^−2^) showed substantially lower average stomatal conductances for the period of the intensive field campaign. Therefore, genetic differences in the control of stomatal opening were an important factor that determined different F_s_ rates among genotypes. Once stomata are open, VPD is the driving force for F_s_, as illustrated by the strong *F*_s_ – VPD relationships for all genotypes.

In addition to root water uptake and *F*_s_, stem tissue water storage is an important factor in tree water relations (Zweifel *et al*., 2000). The pattern of stem diameter variation in response to the replenishment of water storage has been previously observed for poplars under controlled conditions (Giovannelli *et al*., 2007) and for other species (e.g. Zweifel *et al*., 2000, 2001; Conejero *et al*., 2007; Kocher *et al*., 2012). At our site, the highest shrinking and swelling were observed for the genotype with the highest *F*_s_ (Oudenberg), showing that this genotype used an important fraction of its stem water storage to meet its transpiration demand. Skado had lower *F*_s_ than the other genotypes, while it was the highest yielding genotype of those considered in our study (Broeckx *et al*., 2012). This was consistent with previous measurements of leaf gas exchange and intrinsic leaf water use efficiency (Broeckx *et al*., 2014a) and of carbon isotope discrimination (Verlinden *et al*., 2015) performed during the same period in 2011. Therefore, at our site, Skado had the highest water use efficiency as compared to Oudenberg and Grimminge. In addition, carbon isotope discrimination techniques already showed that *P. deltoides* and *P. nigra* (both male and female parental species of Oudenberg) were less water use efficient than *P. trichocarpa* (female parent of Skado) at an experimental poplar plantation in central France (Dillen *et al*., 2008). During a summer drought at a freely draining site in the United Kingdom, genotype Beaupré (*P. trichocarpa* × *P. deltoides*) was able to maintain its transpiration rate for a longer period than genotype Dorschkamp (*P. deltoides* × *P. nigra*) (Hall & Allen, 1997). In response to prolonged drought, stems of SRC poplar genotypes shrank as trees were unable to refill their stem water storage reserves (Giovanelli *et al*., 2007).

In conclusion, we observed that the SRC poplar of our study, which was not water limited in the year 2011, consumed less water as compared to a reference grassland. Moreover, *E*_c_ contributed for 69% to the total growing season ET. The *F*_s_ scaling approach for the intensive field campaign yielded similar results as the modelling exercise. At tree level, we observed important intergenotypic differences in both *F*_s_ and ∆*D* showing different water use strategies associated with different growth strategies among the three genotypes. For our site, Skado had the highest water use efficiency as compared to Oudenberg and Grimminge. Besides harvestable yield, tree water use should be considered as a key criterion in SRC management through careful genotype selection. More large‐scale experiments combining measurements at leaf, plant and stand level under different rotations are necessary to better understand and quantify the water use of SRC at different scales.
